# 4 days in dry immersion increases arterial wall response to ultrasound wave as measured using radio-frequency signal, comparison with spaceflight data

**DOI:** 10.3389/fphys.2022.983837

**Published:** 2022-11-08

**Authors:** Philippe Arbeille, Danielle Greaves, Laurent Guillon, Richard L Hughson

**Affiliations:** ^1^ UMPS-CERCOM Faculté de Medecine—Université, Tours, France; ^2^ Schlegel-University of Waterloo Research Institute for Aging—Waterloo, Waterloo, ON, Canada

**Keywords:** RF signal, carotid, dry immersion, ultrasound, arterial wall

## Abstract

Recent studies have reported a significant increase in common carotid artery (CCA) intima media thickness, wall stiffness and reflectivity to ultrasound, in astronauts, after six months of spaceflight. The hypothesis was that 4 days in dry immersion (subjects under bags of water) will be sufficient to change the CCA wall reflectivity to ultrasound similar to what observed after spaceflight. Such response would be quantified using the amplitude of the ultrasound signal returned to the probe by the target concerned. [coefficient of signal return (Rs)]. The Rs for anterior and posterior CCA wall, sternocleidomastoid muscle, intima layer and CCA lumen were calculated from the ultrasound radio frequency (RF) data displayed along each echographic line. After four days of DI, Rs increased in the CCA posterior wall (+15% +/- 10 from pre DI, *p* < 0.05), while no significant change was observed in the other targets. The observed increase in Rs with DI was approximately half compared to what was observed after six months of space flight (+34% +/- 14). This difference may be explained by dose response (dry immersion only four days in duration). As a marker of tissue-level physical changes, Rs provide complimentary information alongside previously observed CCA wall thickness and stiffness.

## Introduction

Recent studies have reported a significant increase in common carotid artery intima media thickness and wall stiffness in astronauts, during and after six months of spaceflight (Hughson et al., 2016; Arbeille et al., 2017). These morphological and dynamic changes were assessed by ultrasound using B mode echography and time motion mode (M mode). The same measures (increased intima media thickness or wall stiffness) were also observed in the central circulation (aorta) and peripheral limb arteries (superficial femoral artery) (Arbeille et al., 2017), as well as faster pulse arrival time (Baevsky et al., 2007; Hughson et al., 2016).

In the absence of direct human histological data, it was hypothesized that an increase in thickness and stiffness may be related to (a) blood flow and hydrostatic pressure redistribution induced by zero gravity, (b) cardio-metabolic dysregulation (Hughson et al., 2016; Rudwill et al., 2018) induced, in turn, by the reduction of physical activity (Fraser et al., 2012), an inappropriate nutritional regimen (Sandal et al., 2020) and/or environmental and psychological stress (Strollo et al., 2018; Muid et al., 2019). Further, the increase in thickness and or stiffness may be associated with an architectural or content change in the structure of the vessel wall which could change its capacity to reflect ultrasound. The signal return coefficient (Rs) calculated from the native radio frequency (RF) ultrasound signal provides complementary direct information on wall response (components ?), in the absence of a tissue biopsy.

Due to technical limitations described below, traditional B mode and M mode cannot be used to accurately measure “brightness” or reflectance information, particularly on targets located at different depths along the image.

In commercial devices, brightness is problematic to measure directly. The energy reflected by each target is automatically gain-compensated by the software algorithm to artificially display targets at different depths with equal brightness, for example the near and far walls of the carotid artery. In addition, the sonographer can manually adjust the brightness/gain, confounding comparisons across timepoints. In contrast to these traditional, processed modalities, the coefficient of signal return (Rs) is a brightness intensity measure with no post-processing. Rs is different because it sidesteps the processing algorithms in the manufacturer’s software. It is calculated directly from the native ultrasound signal returned by each target intersected by the ultrasound scan line. Thus the coefficient of signal return can quantify the reflectance of various targets (vessel wall, muscle.) independently which allow to evidence physical transformation (structure content) of each of these (Arbeille et al., 2021). The RF signal has been used previously to measure tissue displacement in the carotid artery walls (Palombo et al., 2012), brain tissue (Rytis et al., 2020) and tissue microstructure after being heated by focused ultrasound (Mehdi et al., 2007; Maleke and Konofagou, 2008). Additionally, the native RF data have been used to describe a relationship between increased stiffness and the reduction in micro-displacement inside the tissue (Maleke and Konofagou, 2008).

Human spaceflight analogs have been used to partially reproduce the effects of spaceflight on various physiological systems. Long term head down bedrest induces a fluid shift towards the head and reduces physical activity, while confinement on earth in small habitats does not induce this cephalad fluid shift, but instead induces similar stress along with inactivity. These analogs show some of the arterial changes observed in spaceflight (Arbeille et al., 2014; Carlo et al., 2015; David et al., 2017; Strollo et al., 2018). Lastly, the dry immersion analog, which consists of having subjects lie under bags of water, invokes a fluid shift due to the pressure of the water on the legs and abdomen. Dry immersion is also an proposed analog for stress and complete inactivity (David et al., 2017; Linossier et al., 2017; Greaves et al., 2021). After spaceflight, the amount of ultrasound energy returned to the probe by the arterial walls (Rs) was significantly higher than preflight. This raised the hypothesis that particles that are highly reflective to ultrasound may be the reason, knowing that calcium is released by the bones in microgravity and may have subsequently entered the vessel wall. Knowing that an important bone calcium occurs after only four days in dry immersion (Linossier et al., 2017), we aimed to measure the Rs after 4 days of immersion.

In this study, subjects underwent four-days of dry immersion. We hypothesized that a) common artery vessel wall coefficient of signal return (Rs) would increase due to structural/content changes to the arterial wall, b) this change would be attenuated compared to six months of spaceflight and c) surrounding tissue targets’ coefficient of signal return (Rs) would not increase.

Our objective was to collect and process the RF signal along dedicated, B mode ultrasound scan lines, select the location of each scan line from the B mode image and calculate the coefficient of signal return (Rs) of each of the structures intersected by the scan line. The targets were: sternocleidomastoid muscle, anterior and posterior common carotid artery walls and their respective intima layers, and the lumen.

Data were collected from participants after four days of dry immersion (DI-4days) and compared to the results of another study performed on astronauts (postflight) collected with identical hardware and signal processing methodology (Arbeille et al., 2021).

## Material and methods

### Dry immersion study protocol

A total of 9 subjects were included in the study (9M, age: 34 ± 7, height: 176 ± 6 cm, weight: 74 ± 7 kg). All subjects were informed about the experimental procedures and gave their written informed consent. The experimental protocol conformed to the standards set by the Declaration of Helsinki and was approved by the local Ethics Committee (CPP Est III: 2 October 2018, n° ID RCB 2018-A01470-55) and French Health Authorities (ANSM: 13 August 2018). Clinical Trials.gov Identifier: NCT03915457. The dry immersion protocol included four days of ambulatory baseline measurements before immersion (DI-4days to DI-1day) 5 days (120 h) of dry immersion (DI-1day to DI-5days) and 2 days of ambulatory recovery (R0, R+1). The DI protocol was as previously detailed in De Abreu et al, (2017). During DI, subjects remained immersed in a semi recumbent position for all activities and were continuously observed by video monitoring. Due to various measurements requiring the subjects to move out of the water tank on Day 5, the final ultrasound scans in real DI were completed on day four (DI-4days).

### Spaceflight study protocol

Data from the Vascular Echo study was used to compare dry immersion to spaceflight. Eight subjects (8M) were investigated before and four days following a six month mission to the International Space Station (Arbeille et al., 2021). Crewmembers were tested first thing in the morning, caffeine restricted and prior exercise was controlled. Crew were recruited and all procedures were conducted in accordance with procedures approved by the University of Waterloo Institutional Clinical Research Ethics Board (CREC), NASA JSC Ethics Board (IRB), NASA Human Research Medical Research Board (HRMRB), European Space Agency Medical Research Board (ESA MRB) and the Japanese Space Agency Medical Research Board (JAXA MRB). Identical hardware and processing was completed as for the dry immersion dataset.

### Coefficient of signal return “Rs” evaluation

**FIGURE 1:**
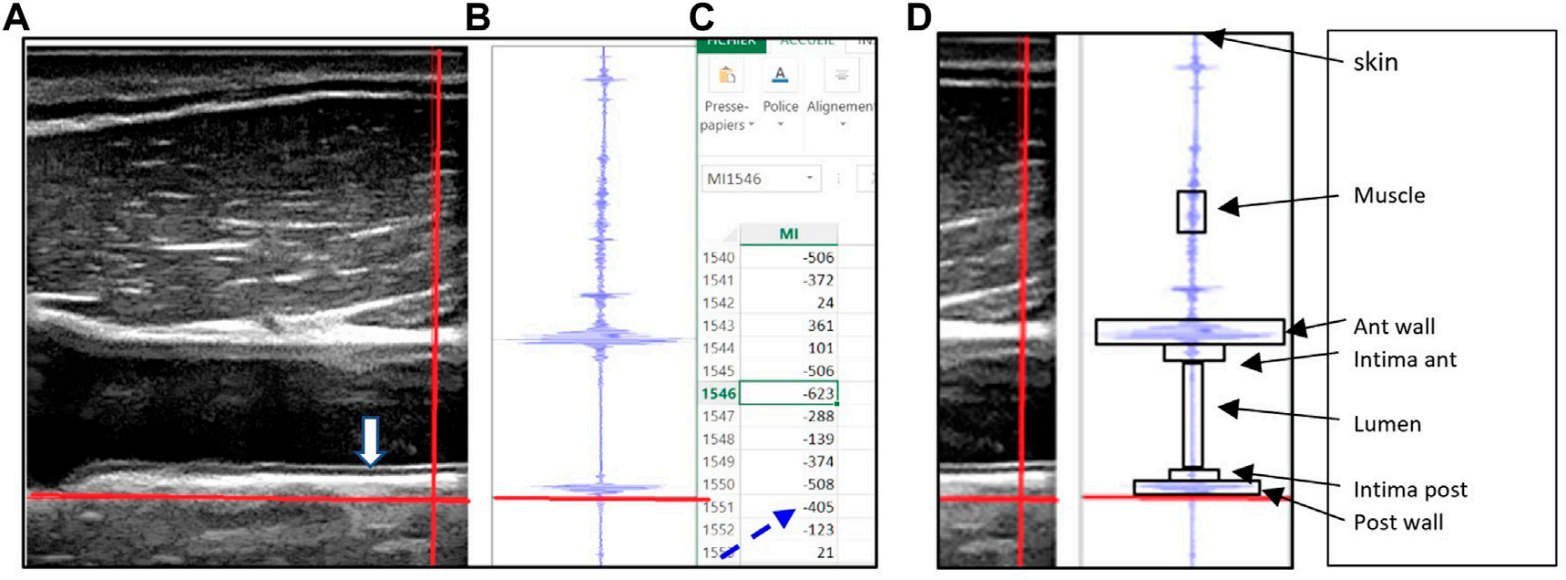
**(A)** B mode echo image with the movable (red) crosshairs intersecting all of the following structures: the sternocleidomastoid muscle, common carotid artery intima layers (anterior and posterior), lumen and artery wall (anterior and posterior), **(B)** the radiofrequency signal displayed graphically for points along the vertical (red) crosshair selected on the B mode image, along with the horizontal red line which allows the user to select the reflected signal value corresponding to the crosshairs’ intersection point. **(C)** Spreadsheet outputs showing the actual reflected signal values, **(D)** Each target is limited by a box (arrows) which allow to identify on the Excel file all the group of cells representing the RF value reflected by the target.

The Sonoscanner echograph (Orcheo-Lite, Paris, France) was customized by the manufacturer to provide the RF data. The Sonoscanner viewer software split the screen vertically into three panels (Figure 1). The B mode image (Figure 1A) provided the user a display of the organs (Carotid) so that the vertical line used for RF output (bright thick line) can be chosen by the operator on this image. In the second panel (Figure 1B), the RF signal intensity trace of the chosen line (on the B mode) was displayed. In the right panel (Figure 1C), an interactive Excel spreadsheet was displayed, listing RF reflected signal amplitude values in a highlighted cell corresponding to the cross section of the vertical and horizontal bright line on the B mode image, as previously described in Arbeille et al, (2021).

To collect the data, the 17 MHz linear scanning probe was placed consistently by an expert sonographer directly on the dry immersion subjects and on the astronauts.

In both studies the probe had to be placed on right side of the neck, long axis orientation, cable end exactly perpendicular to the skin, with one edge in contact with collarbone. Common Carotid Artery (CCA) intima layers (anterior and posterior both) were brought into focus and the RF capture mode over the whole image was triggered.

Offline, five RF sample lines were selected where the six target structures of interest were clearly resolved (box—Figure 1D), and the embedded spreadsheet outputted. The RF signal values were squared (because Amplitude^2^=energy) then summed over the cells (Figure 1C) corresponding to the target area (box along the RF line - Figure 1D), to calculate the energy reflected by each target structure (S target). The energy reflected by these targets were labeled: S. skin, S. muscle, S. anterior wall; S. anterior intima, S. Lumen, S. posterior intima, and S. posterior wall.

The coefficient of reflectivity R was calculated for each of the structures crossed by the ultrasound beam as the ratio between the energy “S(i)” reflected by the structure (i) by the incident energy received by the structure. The incident energy received by each structure (i) is equal to the total incident energy minus the energy reflected by the structures above the present one as previously described in Arbeille et al, (2021). One may notice that the coefficient of signal return is different from the usual “coefficient of reflection” as this one takes into account the energy reflected by the interface between 2 structures while the “coefficient of signal return” takes into account the energy reflected by the whole structure below the interface.

$$R\;muscle=\left(\frac{S\;muscle}{S\;total-S\;skin}\right)$$

$$R\;anterior\;wall=\left(\frac{S\;anterior\;wall}{S,total-S\;muscle\;to\;skin}\right)$$

$$R\;anterior\;intima=\left(\frac{S\;anterior\;intima}{S,total-S\;anterior\;wall\;to\;skin}\right)$$

$$R\;lumen=\left(\frac{S\;lumen}{S,total-S\;anterior\;intima\;to\;skin}\right)$$

$$R\;posterior\;intima=\left(\frac{S\;posterior\;intima}{S,total-S\;lumen\;to\;skin}\right)$$

$$R\;posterior\;wall=\left(\frac{S\;post\;wall}{S,total-S\;posterior\;intima\;to\;skin}\right)$$

In the present example the horizontal red line is located at the lower limit of the posterior wall (on B mode and RF trace) and the corresponding RF reflected value is 405 (dotted arrow). (Figure reproduced with permission from Ultrasound Med biol).

Statistical analysis: A 2-way RM ANOVA (Anova test function, R Studio, Netherlands) was completed to compare the mean coefficient of signal return between the two timepoints (pre DI and at 4 days in DI) among the tissue types, after testing for normality (qqplot). Pairwise t-tests were used for post-hoc comparisons between timepoints with significance at p < 0.05.

**FIGURE 2:**
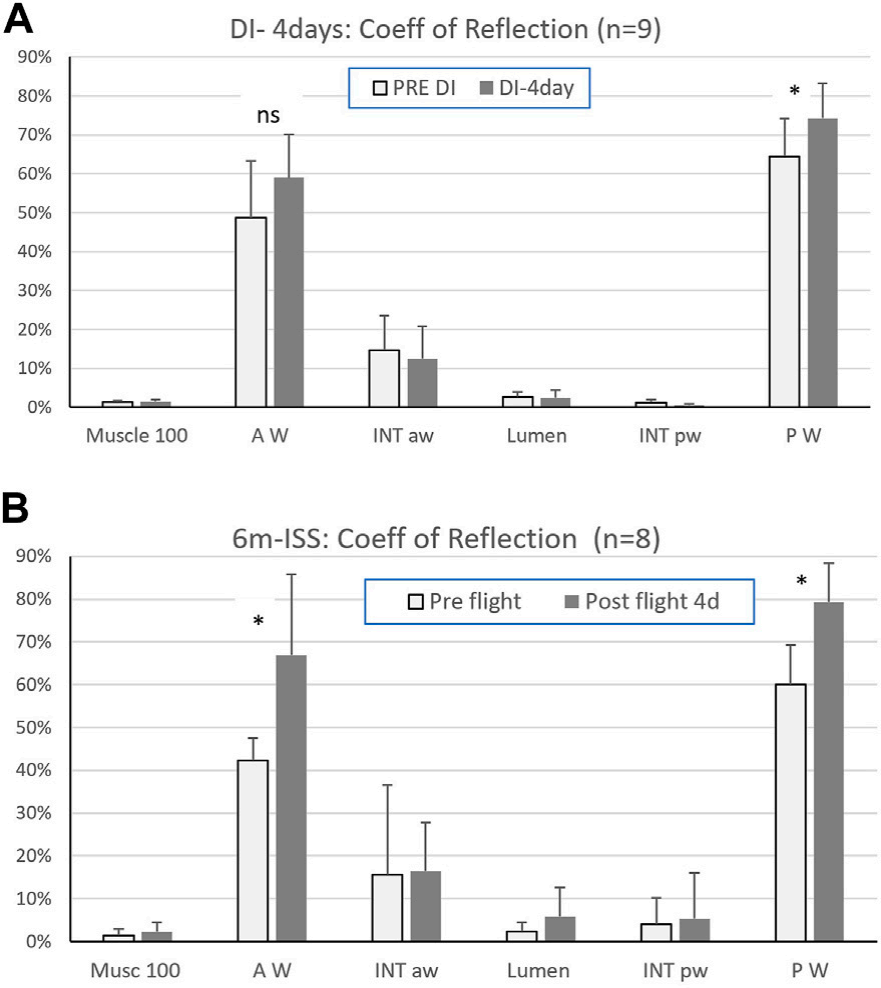
**(A)** Coefficient of signal return of the sternocleidomastoid muscle (Musc 100), carotid artery walls (anterior = AW, posterior = PW), carotid artery intima layers (anterior = INT aw, posterior = INT pw), and lumen, pre and after four days in dry immersion (Mean ± SD - (*) *p* < 0.05; n=9) and **(B)** after six months in space (6m ISS). (Mean ± SD - (*) *p* < 0.05; n=8). (reprinted with permission from Ultrasound Med Biol)

**FIGURE 3:**
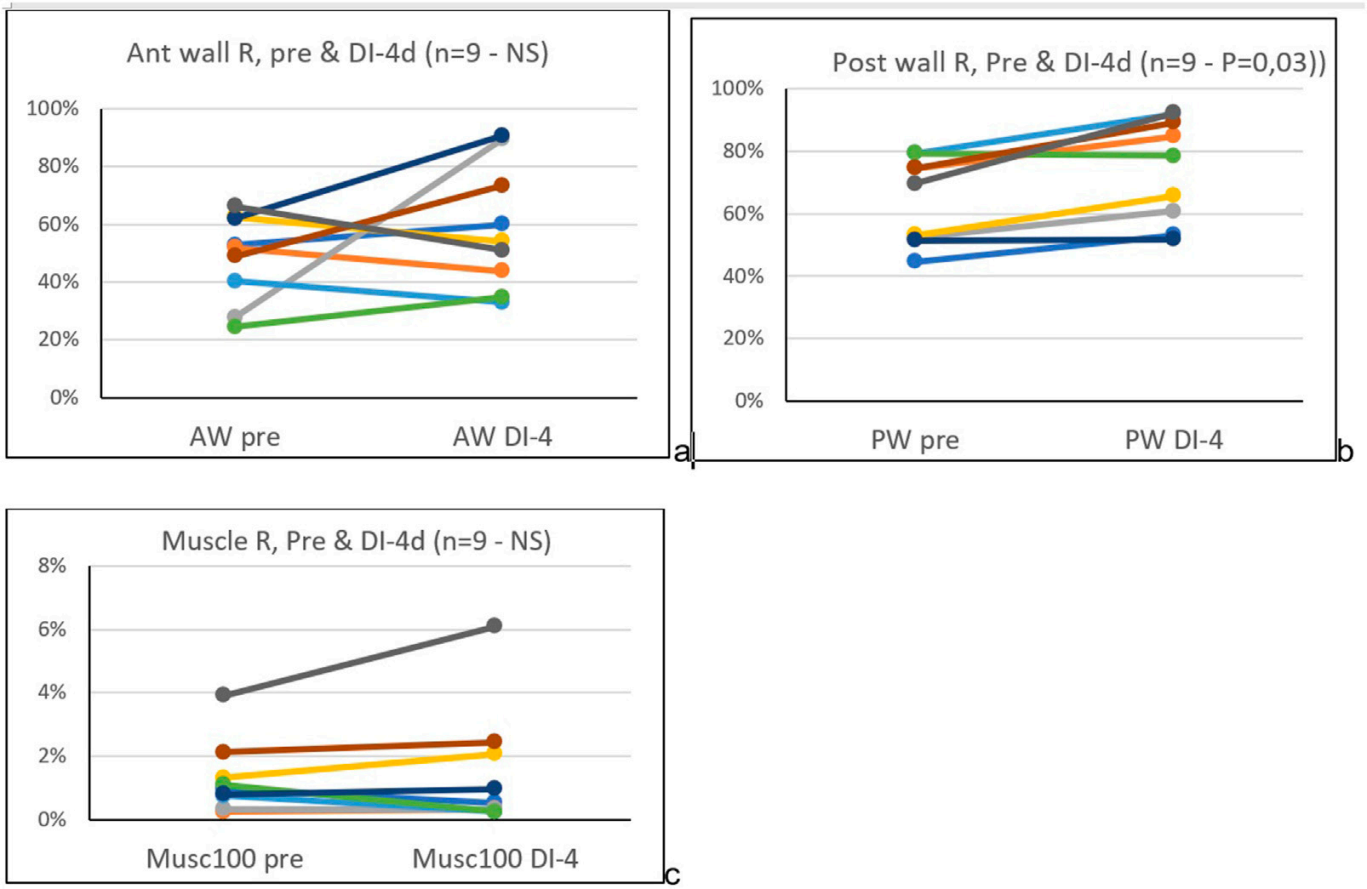
Pre and four days dry immersion, coefficient of signal return (Rs), of the common carotid artery anterior **(A)** and posterior **(B)** wall, and sternocleidomastoid muscle **(C)**, individual data.

**Results**: After four days of dry immersion (DI-4days) the coefficient of signal return of the posterior wall (Figure 2A) was significantly higher compared to pre-immersion (64.4% +/-14 pre to 74.3% +/-16.6, p=0.03; df=8). This corresponded to a mean change of 15% +/-from pre DI. All other targets (muscle, intima layer, lumen) were unchanged (Figure 2A, Figure 3).

## Discussion

After four days in DI and after six months in space, a significant increase in the posterior wall coefficient of signal return Rs was reported, but with a much lower percent increase in DI (15+/-10% in DI versus 33% +/14 postflight). Interestingly this amount of change is in the same order of magnitude as the previously observed increase in stiffness (distensibility index: 9+/-17% (Greaves et al., 2021) and also in spaceflight twice more (distensibility index: 34+/-42%) (Arbeille et al., 2017).

During DI, both carotid arterial wall return ultrasound signal amplitude and stiffness were increased to a lesser extent than after spaceflight (Hughson et al., 2016; Arbeille et al., 2017). The intima media thickness did not change in the DI subjects (Greaves et al., 2021) while it was found to increase in a majority of astronauts (>10% on 5/8) tested (Arbeille et al., 2017). These differences might be dose-related; the dry immersion was short compared to the duration of spaceflight while presenting a high degree of similarities with spaceflight condition: no physical activity, environmental stress, and a significant fluidshift towards the head.

Human histological data on carotid wall structural changes do not exist, but the macroscopic wall modification observed (thickness, stiffness.) in normal subjects in extreme environment (confinement, bedrest, spaceflight, dry immersion) resemble to those observed with aging. Several studies on the elderly population person or Diabetic patients suggest that the aged artery should be characterized by: changes in microRNA expression patterns, autophagy, smooth muscle cell migration and proliferation, collagen and elastin transformation/proliferation and arterial calcification with progressively increased mechanical vessel rigidity and stiffness (Shirwany and Zou, 2010; Tesauro et al., 2017) and reflectance to ultrasound.

Other studies suggest that vascular calcifications are actively regulated biological processes associated with crystallization of hydroxyapatite in the extracellular matrix and in cells of the media or intima of the arterial wall (Lanzer et al., 2014).

If these unknown “products” are highly reflective particles the coefficient of ultrasound signal return should increase. We speculate that these products could be calcium deposits, as increased calcium mobilized from the bone loss into the bloodstream is known to occur with spaceflight (Smith et al., 2015; Vico and Hargens, 2018). In the case of dry immersion, increased bone resorption while evidenced (Linossier et al., 2017) may not be sufficient to explain the increase in signal return coefficient (Rs). But one may notice that the significant loss in bone calcium was the only disturbance present as soon as the first 48h in DI. On the other hand, lower limb arterial calcification with vascular aging (as measured by HR-pQCT has been associated with abnormal bone microstructure and mineral density changes. Such findings suggest a possible pathophysiological link between osteoporosis and vascular calcification (Paccou et al., 2016). Additionally, DI has been shown to impact iron metabolism with increased spleen iron concentration, increased hemolysis, myolysis and serum iron concentration (Kévin et al., 2020). Thus iron deposits may also contribute to the observed increase the Rs coefficient. Without direct histological evidence, however, these processes remains speculative.

Rs represents a ratio between the ultrasound energy returned by the carotid wall and the energy of the ultrasound energy reaching it. The amount of ultrasound energy return depends on the vessel wall cell architecture and content at rest, while the stiffness index is a global mechanical parameter which measures the capacity of the vessel wall to expand during systole i.e. a parameter of function while the artery is not at rest.

The coefficient of signal return provide physiological information for individual layers in the vessel wall as it does for the six separate targets presented here. When combined with a stiffness index, together Rs and distensibility provide a powerful tool to assess both structural (artery at rest) and functional (dynamic artery) changes with spaceflight and aging.

In this paper, the target presented as “wall” is actually the media and adventitia combined and compared separately to the intima layer. For the first time, we present ultrasound data specifically on the media-adventitia, with observed changes in Rs, separately from intima, with no change. This is useful structural information to have because the traditional stiffness measures observe only the global dynamic response of the entire vessel wall to intra-vascular pressure changes. These data show that the increase in stiffness corresponds to change in the media-adventitia layer with no change in the intima.

Arterial stiffness increases with natural human aging (Gepner et al., 2014). A 15% increase in stiffness observed postflight corresponds to, on average15-20 years of aging on Earth. Increased stiffness reported during (after 15days inflight) and after six months of spaceflight (Arbeille et al., 2017) and bedrest were associated with postflight, and post bedrest altered glucose metabolism (Heer et al., 2014; Hughson et al., 2016). but the link between stiffness and cardio-metabolism is not yet clear. Moreover, the trajectory of post-flight recovery is still under evaluation, but even so, it is important to understand the process underlying such a change with real or simulated microgravity in order to develop countermeasures to prevent it during longer sojourns to the moon and Mars.

Contrary to arterial stiffness, we do not have any longitudinal data on the changes in carotid coefficient of signal return (Rs) with natural human aging. Nevertheless, the present study provides value in that it demonstrates that the Rs coefficient changes as early as four days in dry immersion, which means that the vessel wall (structure or content) can modify very quickly and that these changes can be measured by the ultrasound energy returned by the wall.

Importantly, in both studies, the carotid wall Rs coefficient changed against a background of no change in the surrounding tissues, such as the sternocleidomastoid muscle and vessel lumen. These results are consistent with the MRI investigations performed several days post flight (Mc Namara et al., 2019) and at the end of DI, which reported no significant changes to skin or muscle structure and hydric content. These consistent observations point to specificity of the coefficient of signal return as a tissue biomarker.

Study limitation: The dry immersion modality only partially mimics the spaceflight condition. a) While the water bag pressure induces a fluidshift toward the head there is still a 1G gravity applied from the head to the abdomen which partially counteracts this DI fluid shift, especially at the brain level. b) Moreover, during the first day in DI there is a major loss of fluid *via* the kidneys, the subject becomes hypovolemic and the neck/head liquid engorgement reduces significantly by the end of Day 1 (De Abreu et al., 2017). Conversely during spaceflight the neck/head liquid engorgement is present during the entire flight. c) DI does not allow for any physical activity, while during spaceflight the crewmembers have access to exercise equipment such as the T2 treadmill, C2 cycle and ARED resistance device. d) The DI subjects are highly stressed and psychlogicaly affected. e) The number of subjects is limited (n=8 in space and 9 in DI) both in flight and in DI, thus further studies are necessary to confirm the present findings. f) At last the Rs parameter should be validated *in vitro* and/or cadavers using to better understand if calcium particles are possiblyincreasing the Rs. A very basic proof-of-concept *in vitro* study was conducted using the same imaging protocol as above. 2.5g of calcium carbonate was suspended in 20 ml of water (12.5% Ca) and this “phantom” was insonated with the same ultrasound probe as used for DI and spaceflight. The total energy backscattered increased by 17% compared to a plain water control “phantom”. We postulate that this very basic exercise provides further evidence that calcium is reflective and a change in reflection coefficient is possible with calcium in suspension. This result need to be confirmed in a larger study.

In summary, when compared to spaceflight, DI is a model with lower fluid shift concomitant with a higher hypovolemia, a total absence of physical activity and higher stress levels due to complete immobility over several days, and lower calcium release in the blood circulation in relation to bone loss.

## Conclusion

The results support the hypothesis of arterial wall remodeling and changes to wall content, observed after both a short duration space flight analog (4 days DI) and long duration (6 m) spaceflight. Moreover the coefficient of signal return as evaluated from the radio frequency (RF) signal provides information on the physical status (structure/content) of the tissues as early as after 4 days exposure to extreme environment which is not the case with other conventional ultrasound parameter like wall thickness or stiffness. This parameter will be tested on subjects in different space analog environments (ie bedrest/confinement) allowing for countermeasures like physical activity or reduced environmental stress.

## Data Availability

The original contributions presented in the study are included in the article/supplementary material, further inquiries can be directed to the corresponding author.
